# Association between Parkinson disease and selenium levels in the body: A systematic review and meta-analysis

**DOI:** 10.1097/MD.0000000000037919

**Published:** 2024-04-26

**Authors:** Quanyi Chen, Xiaofei Hu, Ting Zhang, Qianying Ruan, Hongye Wu

**Affiliations:** a Department of Clinical Laboratory Medicine, Southwest Hospital, Army Medical University, Chongqing, China; b Department of Nuclear Medicine, Southwest Hospital, Army Medical University, Chongqing, China; c Department of Emergency Medicine, Southwest Hospital, Army Medical University, Chongqing, China; d Department of Blood Transfusion Medicine, Southwest Hospital, Army Medical University, Chongqing, China.

**Keywords:** cerebrospinal fluid, meta-analysis, Parkinson disease, selenium, trace elements

## Abstract

**Background:**

Parkinson disease (PD) is a common neurodegenerative disorder, but its pathogenesis is still not entirely understood. While some trace elements, such as selenium, iron, and copper, are considered pivotal in PD onset due to their role in oxidative stress, the association between selenium concentrations and PD susceptibility remains ambiguous.

**Methods:**

A systematic review and meta-analysis was conducted in adherence to the Preferred Reporting Items for Systematic Reviews and Meta-Analyses guidelines and framed by the Patient, Intervention, Comparison, Outcome paradigm. Data were sourced from 4 prominent electronic databases: PubMed, Embase, Web of Science, and Cochrane Library. Eligible studies must have had a PD case group and a control group, both of which presented data on selenium concentrations. The quality of the studies was assessed using the Newcastle-Ottawa Scale.

**Results:**

Of 1541 initially identified articles, 12 studies comprising a total of 597 PD cases and 733 controls were selected for the meta-analysis. Pronounced heterogeneity was observed among these studies. When assessing blood selenium levels, no significant difference was found between patients with PD and the controls. However, when examining the cerebrospinal fluid, selenium levels in PD patients were significantly elevated compared to controls (standard mean difference = 1.21, 95% CI 0.04–2.39, *P* < .05). Subgroup analyses, sensitivity analyses, and evaluation of publication bias were performed to ensure data robustness.

**Conclusions:**

Elevated selenium levels in cerebrospinal fluid may be associated with a higher risk of Parkinson. Further prospective research is required to solidify this potential link and to offer avenues for novel therapeutic interventions or preventive measures.

## 1. Introduction

Parkinson disease (PD) is a leading neurodegenerative disorder that ranks immediately after Alzheimer disease in terms of its global prevalence. Fundamentally, PD is characterized by the pronounced degeneration and eventual apoptosis of dopaminergic neurons within the substantia nigra pars compacta of the midbrain. Such neuropathological changes culminate in marked depletion of dopamine within the striatum.^[1,2]^ Clinically, it manifests as a triad of resting tremors, heightened muscle rigidity, and postural instability. As the global population skews toward an older age demographic, the epidemiological burden of PD is set to surge, accentuating its socioeconomic and healthcare implications.^[3,4]^

Although the biomedical community has made significant strides in understanding the intricacies of PD, its precise pathogenesis remains an enigma. In the complex landscape of PD pathogenesis, selenium’s role emerges as both a modulator of oxidative stress and an influencer of neuroinflammation. Selenium, through its incorporation into glutathione peroxidase, combats oxidative stress, thereby potentially mitigating dopaminergic neuron degeneration, a hallmark of PD.^[5]^ Concurrently, selenium’s impact on inflammatory pathways, highlighted by its regulation of cytokine production, aligns with emerging research on PD’s inflammatory underpinnings, including the notable roles of neutrophil-to-lymphocyte ratio (NLR) and platelet-to-lymphocyte ratio (PLR) as markers of neuroinflammation.^[6,7]^ This dual capacity underscores the significance of selenium in the neurodegenerative process and supports the investigation into its levels in PD patients.^[8]^ Furthermore, diagnostic criteria for PD emphasize clinical symptoms and advanced diagnostic tools, underscoring the importance of distinguishing PD from other neurodegenerative disorders through biomarkers and neuroimaging, reflecting the multifaceted nature of PD diagnosis and the potential of inflammation-focused research to illuminate novel therapeutic targets.^[9,10]^ Nevertheless, burgeoning research posits an intriguing hypothesis that perturbations in trace element equilibrium could be pivotal in the initiation and progression of PD. Discrepancies in the homeostatic levels of trace elements, notably selenium, iron, and copper, may precipitate oxidative stress, a known antagonist to neuronal integrity.^[11,12]^

Among the myriad trace elements, selenium is an indispensable micronutrient integral to a plethora of physiological processes. The inherent antioxidative mechanisms of selenium are pivotal in mitigating oxidative stress, a well-documented precipitant of cellular degeneration and aging. In addition, selenium is instrumental in modulating immune responses and plays a salient role in both the humoral and cellular arms of immunity.^[13]^ Given its multifunctional nature, selenium has been subjected to rigorous scrutiny in biomedical research. Notably, its potential association with neurodegenerative conditions such as PD has been a focal point. However, the terrain is fraught with ambiguities. Several investigative endeavors delving into comparative selenium concentrations in PD-afflicted individuals versus their non-PD counterparts have yielded incongruent results. Some studies advocate the protective efficacy of selenium against PD pathogenesis, while others refute this association or indicate potentially deleterious implications.^[14,15]^ Such disparities emphasize the importance of comprehensive, methodologically robust investigations to elucidate the precise interplay between selenium concentrations and PD susceptibility.

In our study, while acknowledging the foundational insights from Adani et al^[15]^ and Zhang et al^[16]^ on the role of selenium in PD, we differentiate through extensive subgroup and sensitivity analyses, refining the understanding of selenium’s impact. Our comprehensive approach, incorporating a wide array of data sources, further elucidates the heterogeneity observed across studies and underscores the intricate relationship between selenium levels and PD. This detailed analysis adds novel dimensions to the discourse, highlighting potential therapeutic implications of selenium in PD management not previously explored. Therefore, understanding the association between selenium levels in the body and the risk of Parkinson is of paramount importance. As selenium plays a crucial role in various biochemical pathways, deciphering its association with PD may pave the way for novel therapeutic interventions or preventive measures. This study aimed to ascertain the correlation between selenium levels in the body and the risk of developing PD, thereby enhancing our understanding of this potential interplay and its implications for clinical practice.

## 2. Materials and methods

### 2.1. Search strategy

Throughout our meticulous systematic review and the ensuing elucidation of findings, we strongly abided by the tenets outlined in the Preferred Reporting Items for Systematic Reviews and Meta-Analyses (PRISMA) guidelines.^[17]^ The meta-analytic framework was underpinned by the patient, intervention, comparison, outcome (PICO) paradigm, delineating: patient (P), individuals with PD. Intervention (I): Se levels in the body (either through dietary intake, supplementation, or inherent body concentrations). Comparison (C): Patients not diagnosed with PD. Outcome (O): PD in relation to selenium levels.

Our literature search embarked on July 19, 2023, and encompassed 4 pivotal electronic databases: PubMed, Embase, Web of Science, and the Cochrane Library. Notably, our search was unfettered due to temporal restrictions. The search scheme was orchestrated around pivotal terms including, but not limited to, PD, selenium, dietary selenium, selenium supplementation, and neurodegenerative disorders. These terminologies were judiciously chosen, cognizant of the vast expanse of the PICO paradigm, to ensure a thorough extraction of germane studies for this meta-analysis. Moreover, the bibliographies of pertinent articles were used to mine any further relevant records.

### 2.2. Inclusion criteria and exclusion criteria

During our systematic review and meta-analysis, specific criteria were established to ensure the quality and relevance of the selected studies. In terms of inclusion, eligible studies needed to have a case group consisting of individuals diagnosed with PD, whereas the control group comprised individuals free from PD or any other neurodegenerative disorders. It is imperative that both groups present data on selenium concentrations in the body. Furthermore, the study must have provided data that were readily extractable, which included details on the sample size, mean, and standard deviation of selenium levels, or any data from which these metrics could be discerned. On the flip side, several exclusion criteria were set studies that were literature reviews or had research designs divergent from our focus, publications that appeared to be repetitive presentations of the same research, and any research that was assessed and categorized as being of inferior quality.

### 2.3. Data extraction

A stringent and methodical literature evaluation protocol was established for the meta-analysis. Two independent assessors were responsible for literature screening and subsequent data extraction, ensuring rigorous cross-validation of their findings. In instances where discrepancies emerged, the assessors convened for a deliberative dialogue aimed at rectifying incongruence. If the consensus remained elusive, the expertise of a third impartial reviewer was solicited. The cadre of data points targeted for extraction encompassed author names, publication year, sample sizes of both case and control groups, age demographics, sex distribution, selenium levels, type of specimen used, and method of detection. In scenarios where pivotal data remained absent or opaque in the public domain, proactive outreach was initiated, with the original study investigators contacted via electronic correspondence, soliciting the indispensable, yet unpublished data.

### 2.4. Quality assessment

In the course of our meta-analysis, 2 autonomous evaluators performed a stringent assessment of the quality of the integrated studies using the Newcastle-Ottawa Scale (NOS).^[18]^ NOS, a validated instrument, systematically appraises studies based on a triad of pivotal dimensions: selection, comparability, and outcome, distributed across 9 distinct criteria. This meticulous evaluation facilitates in-depth probing of potential biases intrinsic to the research articles. After this exhaustive assessment, each study was bequeathed with a quality score ranging from 0 to 9. The interpretative metric for these scores is delineated thus: research articles securing scores between 0 and 3 are classified as low caliber; those accumulating scores in the bracket of 4 to 6 are deemed to possess intermediate quality; and manuscripts garnering scores from 7 to 9 are elevated to the echelon of high-quality scholarly contributions.

### 2.5. Statistical analyses

For our meta-analysis, inter-study heterogeneity was scrutinized using the chi-squared test and quantified using the *I*^2^ statistic. A scenario wherein the *I*^2^ statistic remained below 50% accompanied by a *P* value ≥ .10 was interpreted as a lack of notable heterogeneity, prompting the application of the fixed-effects model for consolidated effect size determination. Conversely, an *I*^2^ statistic of ≥50% or a *P* value of <.10 was indicative of pronounced heterogeneity. Under such heterogeneity manifestations, the random-effects model was invoked for amalgamated effect size derivation. In cases of substantial statistical heterogeneity, subgroup analyses were performed to identify and ameliorate potential heterogeneity drivers. The symmetry of the funnel plot was meticulously assessed to appraise the possibility of publication bias influencing the results. A balanced dispersion of data points flanking the funnel plot zenith was illustrative of a diminished propensity for results being swayed by publication bias. To complement this visual assessment, Egger linear regression approach served as a quantitative gauge to detect potential publication bias. All inferential tests were bilateral, and a *P* value threshold of <.05 was established for statistical significance. The analytical procedures were performed using Stata version 17 (StataCorp, College Station, TX, USA).

## 3. Results

### 3.1. Search results and study selection

**Figure 1.**
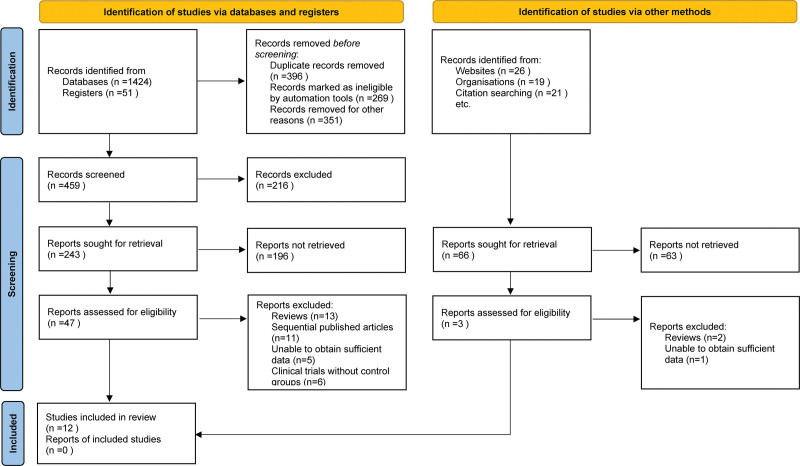
Selection process of included studies.

During a preliminary electronic database query, 1541 pertinent articles were identified. Following the exclusion of duplicates, thorough review of titles and abstracts, and rigorous adherence to the inclusion and exclusion criteria, 47 relevant studies were retained. Of these, 35 were dismissed upon detailed examination, culminating in 12 studies that were finalized for meta-analysis.^[14,19–29]^ A detailed literature filtration trajectory is shown in Figure 1.

### 3.2. Study characteristics

The meta-analysis included 12 studies that evaluated the relationship between selenium levels and PD. These studies, spanning 1995 to 2018, originated from diverse countries such as Spain, India, France, Norway, Iran, the USA, Germany, Sweden, Italy, Tunisia, and China. The combined sample size covered a vast range, from 17 to 238 cases in individual studies. The age brackets of the case groups varied across studies, with some providing precise mean ages and standard deviations, while others offered age ranges of 40 to 80 years. Most studies predominantly utilized serum as the sample type for selenium detection, although other media, such as cerebrospinal fluid (CSF), whole blood, and hair, were also represented. Atomic Absorption Spectroscopy (AAS) and Inductively Coupled Plasma Mass Spectrometry (ICP-MS) were the primary techniques used (Table 1).

**Table 1:** Characteristics of included studies evaluating selenium levels and Parkinson disease.

Author	Year	Country	Sample size (case/control)	Age (case)	Age (control)	Sample type	Detection method
Aguilar et al^[12]^	1998	Spain	18/43	65.5 ± 9.1	65.2 ± 13.0	CSF	CSF
Ahmed et al^[13]^	2010	India	45/42	57.62 ± 9.10	55.62 ± 3.25	Serum	ICP-MS
Baillet et al^[14]^	2010	France	24/30	57.8 ± 8.5	39.4 ± 11.3	Whole blood	AAS
Gellein et al^[15]^	2008	Norway	33/99	40–80	40–80	Serum	ICP-MS
Hemmati et al^[16]^	2017	Iran	40/40	65.7 ± 6.3	64.4 ± 3.8	Serum	AAS
Jimenez et al^[17]^	1995	Spain	29/30	66.3 ± 1.6	67.0 ± 1.8	Serum	AAS
Maass et al^[18]^	2018	Germany	36/42	67.0 ± 11.0	65.5 ± 13.1	CSF	ICP-MS
McIntosh et al^[19]^	2012	USA	23/24	70 ± 9	73 ± 6	Whole blood	ICP-MS
Qureshi et al^[8]^	2006	Sweden	17/21	70.0 ± 15.0	62 ± 11	CSF	AAS
Stefano et al^[20]^	2016	Italy	46/24	72.33 ± 1.25	68.25 ± 1.83	Hair	ICP-MS
Younes et al^[21]^	2013	Tunisia	48/36	65.8 ± 10.2	59.7 ± 12.1	Serum	AAS
Zhao et al^[22]^	2013	China	238/302	66.6 ± 11.3	65.6 ± 12.2	Whole blood	AAS

AAS = Atomic Absorption Spectroscopy, ICP-MS = Inductively Coupled Plasma Mass Spectrometry.

### 3.3. Results of quality assessment

We appraised the methodological rigor of each included study using the NOS. Of these, 3 studies achieved a score of 7, another 3 garnered 8 points, and the remaining 6 were awarded 9 points. None of the studies implemented blinding and no instances of allocation concealment were identified. We detected no bias related to funding sources across studies. Furthermore, there were no studies displaying incomplete outcome data, premature termination bias, or imbalances at baseline. A detailed assessment of bias risks and their associated ratios is presented in Table 2.

**Table 2:** The quality assessment according to Newcastle-Ottawa Scale of each cohort study.

Study	Selection				Comparability	Outcome			Total score
	Representativeness of the exposed cohort	Selection of the nonexposed cohort	Ascertainment of exposure	Demonstration that outcome	Comparability of cohorts	Assessment of outcome	Was follow-up long enough	Adequacy of follow up of cohorts	
Aguilar et al^[12]^	★	★		★	★	★	★	★	7
Ahmed et al^[13]^	★	★	★	★	★★	★	★	★	9
Baillet et al^[14]^	★	★	★	★	★★	★	★	★	9
Gellein et al^[15]^		★	★	★	★★	★	★	★	8
Hemmati et al^[16]^	★	★	★	★	★★	★	★	★	9
Jimenez et al^[17]^	★	★	★	★	★★	★		★	8
Maass et al^[18]^	★	★		★	★	★	★	★	7
McIntosh et al^[19]^	★	★	★	★	★★	★	★	★	9
Qureshi et al^[8]^	★	★	★	★	★★	★	★	★	9
Stefano et al^[20]^	★	★	★	★	★	★	★	★	8
Younes et al^[21]^	★	★	★	★	★★	★	★	★	9
Zhao et al^[22]^	★		★	★	★	★	★	★	7

### 3.4. Meta-analysis on selenium levels in PD patients

**Figure 2.**
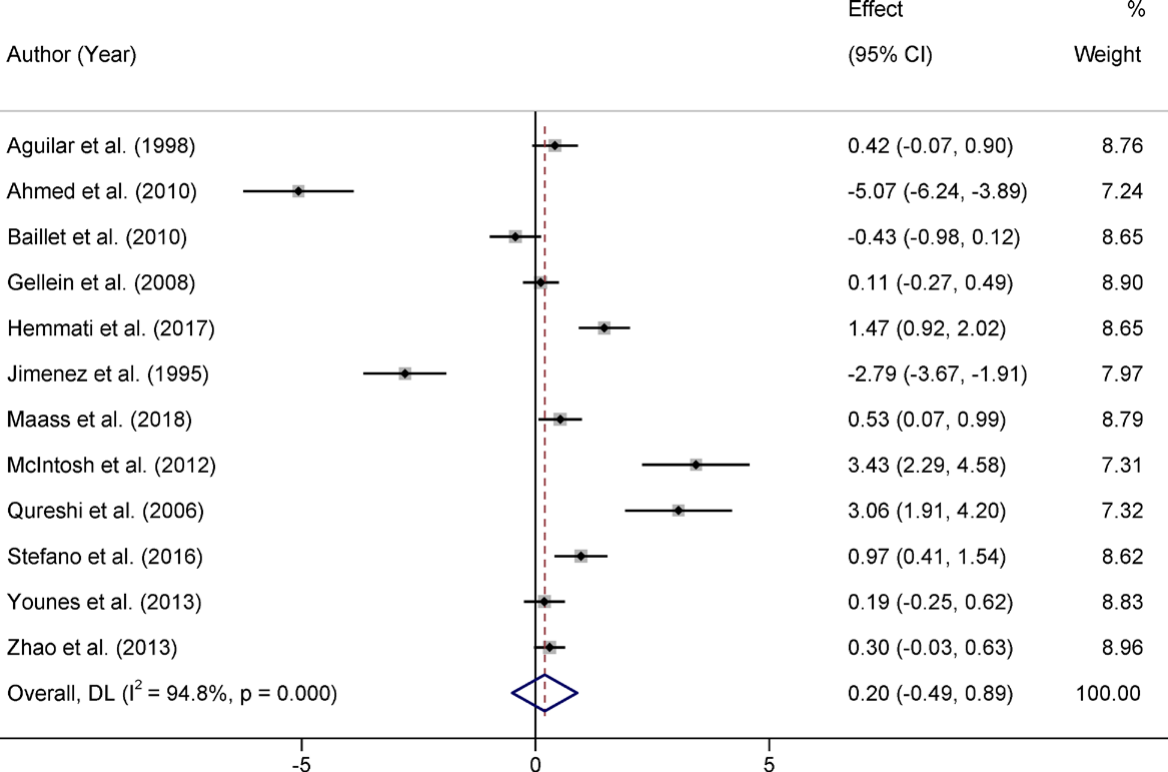
Forest plots of the selenium levels in Parkinson disease patients.

**Figure 3.**
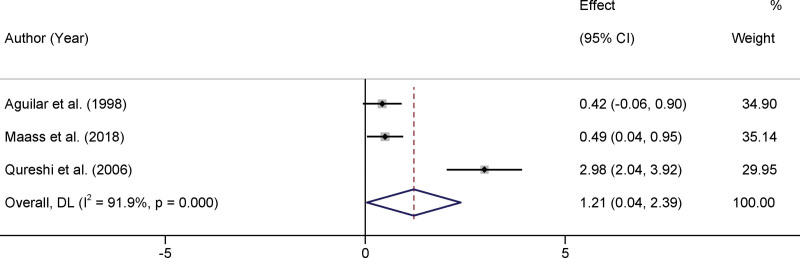
Forest plots of the selenium levels in the CSF of Parkinson disease patients. CSF = cerebrospinal fluid.

In our meta-analysis, we evaluated data from 12 distinct studies that compared selenium levels in the serum or whole blood of patients with PD to control groups. Heterogeneity was prominent among the selected studies, with an *I*² value of 94.8% and *P* < .001, indicating a significant variation in the results across studies. Owing to high heterogeneity, a random-effects model was employed for data amalgamation. After consolidation of the available data, it was determined that there was no statistically significant difference in selenium levels between the PD patients and control groups. The standard mean difference (SMD) was 0.20, with a 95% CI ranging from −0.49 to 0.89 (*P* > .05). The absence of statistical significance suggests that the role of selenium levels in PD has not been conclusively established, and further research is required to understand the underlying mechanisms and their potential implications in disease progression or onset (Fig. 2). Three studies reported selenium levels in the CSF of patients with PD compared with control groups.^[14,19,25]^ Based on the random-effects model, the pooled results demonstrated that CSF selenium levels in patients with PD were significantly higher than those in the control group (SMD = 1.21, 95% CI 0.04–2.39, *P* < .05, Figure 3).

### 3.5. Subgroup analysis

Our meta-analysis further segregated the data into various subgroups to obtain a clearer understanding of the blood selenium levels under different conditions. These subgroups were differentiated based on geographic location, detection method, age matching, sample type, and year of publication. Heterogeneity was particularly high across most subgroups, as indicated by the *I*^2^ values being predominantly above 90%. However, statistical significance varied among these subgroups. Notably, selenium concentrations in the serum and whole blood did not show a consistent trend. The publication year also presented differences, hinting at the potential evolution of study methodologies or population diets over time. It is essential to consider these subgroup variations in order to understand the broader implications of the data (Table 3).

**Table 3:** Subgroup analysis of blood selenium levels in various populations.

Subgroup	Number of studies	SMD (95% CI)	*I * ^2^	*P* value
Geographic location				
Asia	7	−0.22 (−1.45 to 1.00)	98.2%	.001
Europe	4	−0.81 (−2.20 to 0.58)	94.8%	.001
Detection method				
AAS	9	0.10 (−0.70 to 0.90)	95.9%	.001
ICP-MS	3	−1.40 (−5.00 to 2.20)	98.5%	.001
Fluorescence spectroscopy	2	0.43 (−1.36 to 2.22)	91.9%	.001
Age matched				
Yes	9	−1.20 (−2.50 to 0.10)	97.3%	.001
No	4	1.45 (0.16–2.74)	94.5%	.001
Sample type				
Serum	11	−0.50 (−1.70 to 0.70)	97.2%	.001
Whole blood	3	0.90 (−0.45 to 2.35)	94.5%	.001
Publication year				
1995–2009	7	−0.10 (−1.40 to 1.20)	96.5%	.001
2010–2020	6	−0.15 (−1.35 to 1.05)	96.7%	.001

AAS = Atomic Absorption Spectroscopy, ICP-MS = Inductively Coupled Plasma Mass Spectrometry.

### 3.6. Sensitivity analysis

**Figure 4.**
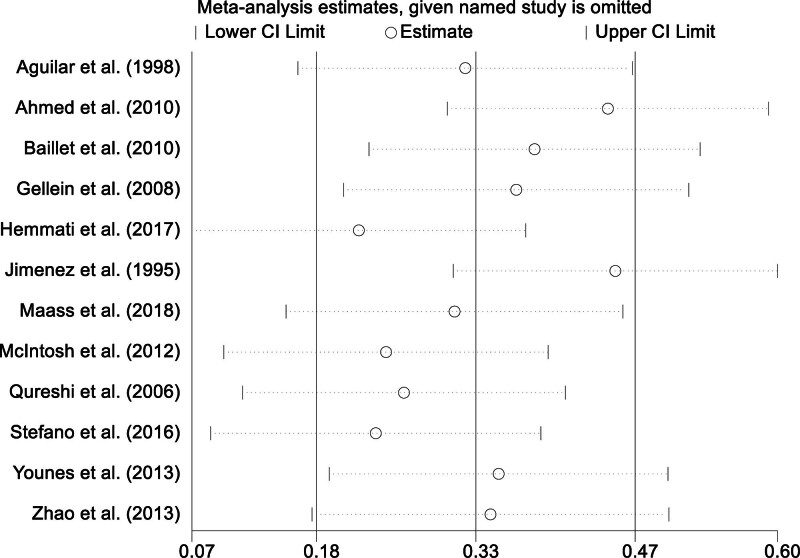
Sensitivity analysis.

In light of the considerable heterogeneity identified across the studies included in our meta-analysis, we performed a sensitivity analysis to evaluate the consistency and trustworthiness of the aggregated outcomes. Each study was systematically omitted, and the effect size was recalculated for residual studies. This meticulous sensitivity evaluation affirmed that the aggregated outcomes were unwavering and remained trustworthy, irrespective of the omission of any particular study. This finding suggests that the overarching conclusions were not disproportionately swayed by any single study, thereby bolstering the credibility of our conclusions. The resilience of the results of these tests reaffirms the solidity of our principal outcomes and further reinforces the inferences made in this meta-analysis (Fig. 4).

### 3.7. Publication bias

**Figure 5.**
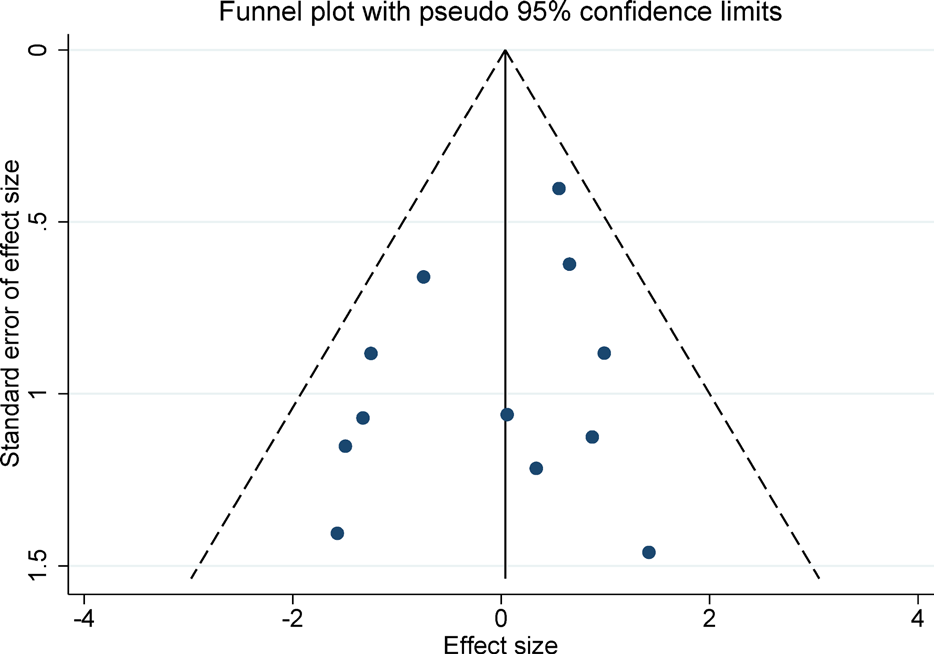
Funnel plot for publication bias.

Upon careful examination of funnel plots constructed from the data of the studies integrated into this meta-analysis, we observed a symmetrical pattern. This symmetrical distribution typically implies an absence of significant publication bias, and this assertion is visually evident in Figure 5. To provide a statistical perspective on potential bias, we employed Egger regression test. The outcomes of this test consistently demonstrated no significant evidence of publication bias across the different variables assessed in our meta-analysis, with all *P* values exceeding .05. These results not only underscore the lack of publication bias but also offer an additional layer of assurance regarding the trustworthiness and stability of our meta-analytical conclusions.

## 4. Discussion

Our comprehensive meta-analysis, adhering to the PRISMA guidelines and utilizing a broad search strategy across major databases, marks a significant endeavor to elucidate the association between selenium levels and PD. The novel findings of this study, particularly the elevated selenium levels in the CSF of PD patients compared to controls, suggest a potential compensatory mechanism in response to oxidative stress, a known contributor to PD pathogenesis. This insight underscores the importance of selenium in the neurodegenerative processes of PD and highlights the potential for selenium modulation as a therapeutic avenue. Additionally, our rigorous subgroup analysis, considering factors such as geographic location, detection method, and sample type, addresses the heterogeneity within the existing literature, offering a nuanced understanding of selenium’s role across different populations and conditions. The clinical implications of these findings could be profound, providing a foundation for future research into selenium-based interventions for PD, with the ultimate goal of enhancing patient care and outcomes in this challenging neurodegenerative disorder.

Se, an essential trace element required for normal human physiology, operates within a narrow therapeutic window. Its importance is underscored by the detrimental health effects linked to both deficiency and overexposure. The distinct biological properties of selenium are largely contingent on its chemical form, whether inorganic or organic. Epidemiological studies have highlighted an increased mortality rate among PD patients exposed to inorganic hexavalent selenium via drinking water compared with those not exposed.^[30,31]^ Dietary selenium is predominantly in its organic form, which undergoes transformation into seleno-compounds upon absorption. These compounds contribute to the synthesis of selenoproteins and glutathione peroxidases, which are pivotal for antioxidant defence mechanisms in the central nervous system.^[32]^ These antioxidant properties could potentially protect dopaminergic neurons from oxidative damage.

However, the dual role of selenium is evident given its potential to exert oxidative stress, among other mechanisms that could negatively impact the central nervous system. There is a growing body of evidence suggesting that excess selenium exposure might detrimentally affect dopaminergic neurons by generating reactive oxygen species and modulating the mRNA expression of dopamine receptors, tyrosine hydroxylase, and dopamine transport genes, thereby paving the way for neurodegenerative changes.^[33]^ Even at minimal selenium concentrations, neurotoxic effects may manifest. Some studies have postulated that selenium can activate the p38 pathway, which has been implicated in the pathogenesis of PD.^[34]^ Our comprehensive meta-analysis, encapsulating the findings from various case–control studies, indicated that while selenium levels in the blood of PD patients were analogous to those in the control group, CSF levels were elevated, and hair levels were reduced. These observations suggest that blood selenium levels may not be directly related to the onset of PD. Conversely, elevated selenium levels in the CSF could be a potential risk factor, while high selenium levels in hair might serve as protective elements against the disease.

Selenium’s role in PD involves its integration into selenoproteins like glutathione peroxidases and thioredoxin reductases, combating oxidative stress crucial in PD pathogenesis.^[35]^ Elevated CSF selenium in PD may indicate enhanced antioxidative pathways, possibly a neuroprotective response against oxidative damage to dopaminergic neurons.^[36]^ This suggests selenium’s broader role in neurodegenerative conditions.^[5]^ Oxidative stress, a common element in parkinsonism, including multiple system atrophy (MSA), progressive supranuclear palsy (PSP), and corticobasal degeneration (CBD), may be mitigated by selenium’s antioxidative role, as seen in PD with elevated CSF selenium levels suggesting an adaptive mechanism.^[37]^ This potential across parkinsonian disorders warrants specific research into selenium’s therapeutic capacity, highlighting the necessity for targeted studies on selenium’s effects in atypical parkinsonisms.^[38]^

A longitudinal study posited that plasma selenium levels might not be inherently linked to the presence of PD but were positively correlated with neurological tasks assessing coordination and motor speed.^[39]^ Furthermore, the relationship between trace element levels in the CSF and their corresponding peripheral and circulating levels is often nonlinear, especially in patients with PD and other neurodegenerative disorders.^[40]^ Such disparities might stem from alterations in metal transporters or blood–brain barrier dynamics, thereby modulating the proportion of metal elements between the blood and CSF. Although tissue-specific selenium levels might not be inherently correlated, discrepancies between inorganic and organic selenium forms have been observed, particularly between blood and CSF samples from healthy subjects.^[41,42]^ Unfortunately, owing to a lack of data pertaining to variations in CSF selenium levels across different stages of PD, we were unable to delve deeper into the mechanisms underlying the observed selenium changes in the central nervous system preceding or following PD onset.

Hair selenium concentrations provide a retrospective window into an individual’s nutritional status over weeks to months. However, these levels can be influenced by various factors such as age, sex, and environmental selenium concentrations. The potential confounders were not rigorously controlled in the 2 primary studies incorporated into our research, possibly affecting the veracity of our results. From a pathophysiological and biological standpoint, for PD patients, the selenium concentration in the CSF, a vital component of the central nervous system and closely related to neurodegenerative diagnosis, might be more insightful than those in the blood or hair.^[43,44]^ This dichotomy in selenium levels suggests that higher selenium levels in the CSF of patients with PD could be indicative of selenium exposure as a risk factor for PD. From a preventive and control perspective, it is paramount to ensure that selenium intake for high-risk groups, especially the elderly, remains within the optimal limits. In our subgroup analysis, we examined selenium levels in PD patients, considering factors like geographic location, detection method, age matching, sample type, and publication year. Despite considerable heterogeneity, no uniform trend in selenium levels between PD patients and controls was observed across subgroups. This variability highlights the complexity of selenium’s role in PD and the influence of environmental, methodological, and demographic factors. Our findings suggest the necessity for further research to clarify selenium’s impact on PD, taking into account these diverse influences.

Recent studies exploring the therapeutic effects of selenium on alpha-synuclein aggregation in the substantia nigra pars compacta offer promising insights.^[8,9]^ Selenium, through its incorporation into selenoproteins, plays a critical role in mitigating oxidative stress, which is implicated in the pathogenesis of PD. The potential of selenium to influence the aggregation of alpha-synuclein, a key pathological feature of PD, highlights its relevance not only as a trace element of interest but also as a possible therapeutic agent.^[45]^ Such findings underscore the necessity of further research to explore selenium’s impact across different stages of PD, potentially unveiling stage-specific therapeutic or preventive measures.^[46]^ This aspect aligns with our findings of elevated selenium levels in the CSF of PD patients, suggesting that selenium may play a complex role in the disease’s pathophysiology that warrants detailed investigation.

This study has several limitations must be acknowledged. First, our reliance on retrospective data introduces a potential recall bias and may lead to gaps in the information collected. Additionally, while the sample size was deemed adequate, it may lack the power to detect more nuanced effect sizes or infrequent outcomes. Furthermore, the homogeneity of our study population limits the generalizability of the findings to a broader demographic group. It is essential to interpret the results in light of these caveats.

## 5. Conclusions

In conclusion, the current research indicates a potential correlation between selenium levels in the CSF and the onset of PD, with elevated selenium in the CSF emerging as a primary risk factor. Future high-quality, prospective research is imperative to confirm elevated CSF selenium as a significant risk factor for PD.

## Author contributions

**Data curation:** Quanyi Chen.

**Formal analysis:** Quanyi Chen, Qianying Ruan.

**Writing – original draft:** Quanyi Chen.

**Investigation:** Xiaofei Hu.

**Methodology:** Xiaofei Hu.

**Resources:** Ting Zhang, Qianying Ruan.

**Software:** Ting Zhang.

**Supervision:** Hongye Wu.

**Validation:** Hongye Wu.

**Visualization:** Hongye Wu.

**Writing – review & editing:** Hongye Wu.
